# An unusual case of gout in the wrist: the importance of monitoring medication dosage and interaction. A case report

**DOI:** 10.1186/1746-1340-15-16

**Published:** 2007-10-09

**Authors:** Craig L Jacobs, Paula J Stern

**Affiliations:** 1 Graduate Education and Research Programs, Canadian Memorial Chiropractic College, 6100 Leslie St., Toronto, ON M2H 3J1, Canada

## Abstract

**Background:**

Gouty arthritis of the wrist is uncommon although gout itself is the most common inflammatory arthritis in older patients. Some known risk factors for the development of gout include trauma, alcohol use, obesity, hyperuricaemia, hypertension and diabetes mellitus. As well, certain medications have been shown to promote the development of gout. These include thiazide diuretics, low dose salicylates and cyclosporine. We present a case of gouty wrist pain possibly precipitated by a medication dosage increase as well as medication interactions.

**Case presentation:**

A 77 year old male presented with right wrist pain. Redness and swelling was present at the dorsal aspect of his wrist and range of motion was full with pain at end range upon examination. One week prior, his anti-hypertensive medication dosage had been increased. The patient's situation continued to worsen. Radiographic examination revealed changes consistent with gouty arthritis.

**Conclusion:**

It is important for clinicians treating joint conditions to be aware of patients' comorbidities, medication usage and changes in dosages. Education of patients with gout is of prime importance. Clinicians should educate patients that gout may occur at any joint in the body not only the lower limb. Patients should be aware of the signs and symptoms of an acute gouty attack and be made aware that changes in certain medication dosages may precipitate an attack. Awareness of radiographic changes associated with gout is still of importance although these changes are not seen as frequently as they have been in the past due to better control of the disease.

## Background

Joint pain accompanied with swelling is a common complaint seen in clinical practice. The challenge is to determine the underlying etiology and to provide the appropriate treatment. Many joint diseases present as acute monoarthritis with the most common causes due to gout or calcium pyrophosphate dihydrate crystal deposition disease (CPPD) [1]. The peak incidence of gout is between the ages of 30–50 with the prevalence increasing with age [2]. Both the incidence and prevalence of gout has been on the rise in recent years [3]. The increased prevalence is believed to be related to several factors which include increased age and obesity in the population and widespread diuretic use for hypertension treatment [3,4].

Gout is five times more common in men. Most acute gouty attacks occur in a single joint in the lower limb with the first metatarsal joint most commonly affected [2,5]. On clinical presentation, the joint often appears red, swollen and very tender. Some differentials to keep in mind include septic arthritis, rheumatoid arthritis, osteoarthritis and erosive arthritis [1]. Some known risk factors for the development of gout include trauma, alcohol use, obesity, hyperuricaemia, hypertension and diabetes mellitus [2,5]. As well, certain medications have been shown to promote the development of gout. These include thiazide diuretics, low dose salicylates and cyclosporine [2,5].

We present an unusual case of gouty wrist pain possibly precipitated by a medication dosage increase as well as medication interactions.

## Case presentation

A 77 year old male was treated at a chiropractic clinic for low back pain resulting from lumbar facet arthrosis and lateral canal stenosis. On a subsequent visit he reported right wrist pain which began while lifting a heavy box. On examination, redness and swelling was noted on the dorsal aspect of his right wrist. Range of motion was full with pain at end range of flexion and extension. His health history included two hip replacements, two previous episodes of gout in both first metatarsophalangeal joints (2 and 5 years prior), and hypertension. Medications for hypertension included perindopril (4 mg), hydrochlorothiazide (25 mg), and Norvasc (10 mg). In addition, he was prescribed 80 mg of aspirin/day and took a daily multivitamin. One week prior, the patient's general practitioner had increased his Norvasc dosage and also prescribed Tylenol 3 to be taken as needed for his back pain.

Two days later the swelling had increased in the dorsal aspect of his right wrist and hand. Wrist flexion was limited by 80% with severe pain. Pain was present on palpation of the scaphoid bone. Due to the suspicion of fracture, the patient was referred to his general practitioner for radiographs. The radiologist who read the films described tiny cysts at the distal radius and concluded that these were most likely due to old trauma. Mild osteoarthritic changes were noted at the carpal-metacarpal joint at the base of the thumb. The report stated that the radiographs were otherwise normal.

**Figure 1:**
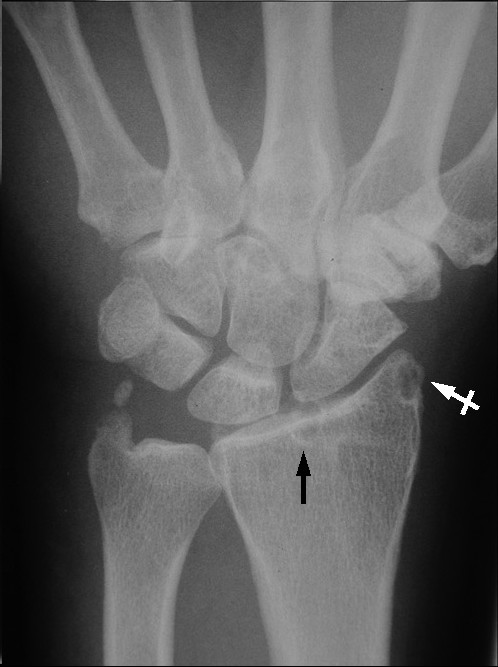
PA view of right wrist reveals a subchondral cyst in the distal radius (black arrow) and a well-marginated juxta-articular bony erosion at the radial styloid process (white crossed arrow).

The patient's symptoms worsened with increased pain and swelling over the next few days. Due to the worsening symptoms, repeat radiographs were performed five days later and were read by a radiologist. The radiographs revealed well-marginated juxta-articular bony erosions at the radial styloid process and the dorsal rim of the distal radius with soft tissue swelling. These findings were deemed to be consistent with gouty arthritis. (See figure 1).

The patient was referred back to his general practitioner with the radiologist's report and the patient was immediately put on colchicine. At follow up two days later, the wrist swelling had decreased significantly. One week after the initiation of colchicine, no swelling was present and only mild pain was noted with flexion/extension of his wrist and fingers. The patient was taken off colchicine due to diarrhea. A recurrence of wrist pain occurred several weeks later. The patient was referred to a rheumatologist who prescribed colchicine for one month duration. When the pain resolved completely, the patient was prescribed allopurinol. Anti-hypertensive medications were not altered. At one year follow up, no further gouty attacks had been reported.

## Discussion

A review of the literature reveals that gouty arthritis of the wrist is rare in isolation although gout itself is the most common inflammatory arthritis in older patients [4,6-9]. Gout at the wrist as the initial appearance of the condition occurs between 0.8 to 2% of all gout cases [9]. Gout patients who are not treated have a 19–30% chance of developing gout in the wrist during their lifetime [9]. Reported cases of carpal tunnel syndrome, tendon entrapment or rupture, and scapholunate dissociation have been reported in the literature due to tophaceous deposits in the wrist due to gout [6,8,9]. The prevalence of gout in the USA ranges between 0.5–2.8% in men and 0.1–0.6% in women [2]. The prevalence rises to 4.4% of men and 1.8% of women over the age of 65 [4]. A two-fold increase in incidence of gout has been reported in the USA and New Zealand over the past 30 years, while the prevalence of gout has been reported to have risen three-fold in the UK over 20 years of follow-up [10]. This rise may be attributed in part to the continuous aging of the population as well as the widespread use of diuretics for treatment of hypertension [4].

Gout is a clinical syndrome caused by the deposition of monosodium urate monohydrate crystals into synovial, bursal, and cartilaginous tissue. The underlying metabolic disorder is hyperuricemia. The exact trigger of an acute attack of gout is poorly understood however predictors for the development of gout in hyperuricemic individuals have been identified. These include: increasing uric acid level, alcohol consumption, hypertension, use of diuretic drugs (thiazides and loop diuretics), increased body mass index, and family history of gout [4,5,11]. These predictors appear to have an additive effect on the risk of developing gout [11].

Hyperuricemia results from either decreased renal excretion (which occurs in 90% of gout patients) or hyperproduction of uric acid [4]. Drugs that may cause hyperuricemia and gout include: diuretics, cyclosporine, low-dose aspirin, ethambuthol, pyrzinamide, and nicotinic acid [4]. As some are commonly prescribed medications, it is imperative that health care practitioners dealing with joint and musculoskeletal conditions be aware of the medications that their patients are taking. In addition, they should be prudent to be made aware of any changes in prescribed dosages. In this case, the patient was taking diuretics as well as other anti-hypertensive medications, aspirin, and vitamin B3 (nicotinic acid).

A retrospective cohort study found a substantially increased risk of receiving treatment for gout among elderly hypertensive patients who were prescribed thiazide diuretics when compared to those subjects who were receiving non-thiazide antihypertensive medications [12]. The thiazide diuretic therapy subjects were almost twice as likely to have undergone anti-gout therapy. This risk increased even more so when thiazide diuretics were combined with any other non-thiazide antihypertensive medication. In this case, the patient's non-thiazide antihypertensive medication was increased. This change in blood chemistry may have contributed to the precipitation of the acute gouty attack however the exact trigger of this acute gouty attack cannot be determined and is most likely multifactorial. The mechanisms by which diuretics contribute to elevated serum uric acid levels are: decreased filtration of uric acid, increased reabsorption, as well as decreased secretion [12]. Ene-Stroescu and Gorbien state that due to these mechanisms, diuretics are the most common cause of secondary gout with diuretic use being reported in over 75% of patients with late-onset gout and even approaching 100% in women [4]. The amount of risk is also related to dosage. Thiazide diuretic dosages less than 25 mg/day did not have a significant increase in risk, whereas dosages ≥25 mg/day had a relative risk of between 2.10–2.16 [12]. In this case, the patient was taking a dosage of 25 mg/day and thus had an increased risk of gouty attack. Gurwitz et al state that low doses of thiazide diuretics can be just as efficacious as larger doses with a reduced risk of metabolic disturbances. Doses as low as 6.25 mg can be effective when combined with another low dose anti-hypertensive medication [12].

Aspirin is prescribed widely in the elderly. Given that aspirin is attainable without prescription, this leads to problems with self-prescription and dosage issues. Low-dose aspirin, up to 2 g/day has the potential to increase uric acid retention [11,13]. The combination of low-dose aspirin and diuretics compounds this effect [13]. Clinicians should inquire regarding aspirin usage in patients due to this widespread and often unmonitored use.

The three clinical stages of gout are: acute gouty arthritis, intercritical gout, and chronic tophaceous gout. Acute gouty arthritis refers to acute inflammation due to the precipitation of urate crystals within a joint. Gouty attacks may be precipitated by trauma, starvation, surgery, ingestion of high purine content food, excessive alcohol intake, and drugs that affect urate concentration [4]. It should be noted that drugs that reduce urate concentration may also precipitate a gouty attack [4]. Initial attacks most commonly occur in the lower limbs and are usually monoarticular with up to 50–60% occurring at the first metatarsophalangeal joint [2,4,14]. Gout may occur in any joint including the ankle, knee, hand, wrist, elbow, sacroiliac joint and other joints of the spine, however most commonly occurs in the lower extremity. In this case, although the patient had suffered two previous gouty attacks in the first metatarsophalangeal joint he was unaware that gout could occur in his wrist.

Typical presentation includes sudden onset of intense pain, redness and swelling of the joint. Examination will reveal a red, swollen, and extremely tender joint. Natural history of an acute attack ranges from a few days to a few weeks. Radiographs during early attacks may only reveal soft-tissue swelling. Serum uric acid levels may be normal during an attack due to pro-inflammatory cytokines [5].

The majority of untreated patients will experience another acute attack within 2 years [4]. Prophylactic treatment is usually recommended in patients who have more than 2–3 gouty attacks per year [5]. Recent studies have advocated the avoidance of diuretics, weight gain and alcohol consumption. A low carbohydrate, high protein and unsaturated fat diet has also been recommended as it enhances insulin sensitivity and may reduce serum uric acid levels [13].

Patients who experience multiple attacks of acute gouty arthritis are predisposed to the development of polyarticular gouty arthritis [14]. Attacks can then occur in more than one joint simultaneously, especially in the lower extremity. This emphasizes the unusual presentation in this case of an isolated attack of gout in the wrist. Acute onset of polyarticular gouty arthritis is more frequently seen in older patients most of whom are receiving diuretics for the management of hypertension [4]. Radiographic findings also tend to lag behind the clinical manifestations of gout by 5–10 years [14]. This is an especially unusual aspect of this case in that the patient had no previous gouty attacks in the wrist and radiographic changes were present during this first acute episode in his wrist.

The success of prophylactic measures has led to a significant decrease in the numbers of patients developing chronic tophaceous gout [14]. Chronic tophaceous gout occurs after years of recurrent acute gouty attacks and is characterized by persistent pain and swelling in the affected joints. Classic radiographic features include soft tissue densities (tophi) and para-articular bony erosions [14]. Joint space is generally well maintained. Subchondral cysts may be present as they were in this case. (See Figure 1) Due to the increasing rarity of these x-ray changes because of better management, it is possible that clinicians may not be as familiar with these changes, especially in the early stages of bone and joint destruction. Radiographs still remain the imaging examination of choice for gouty arthritis although advanced imaging techniques may be used. The appearance of gout in MR imaging is variable. Joint effusion and para-articular edema may be present in an inflamed joint. Tophaceous deposits will appear low to intermediate signal intensity on T1-weighted images and range from low to high signal intensity on T2-weighted images depending on the degree of hydration of the tophi [2].

Differential diagnoses to consider include rheumatoid arthritis, osteoarthritis, septic arthritis, calcium pyrophosphate dihydrate crystal deposition disease, erosive arthritis, psoriatic arthritis, xanthomatosis, and amyloidosis. The definitive diagnosis of gout is made by examination of synovial fluid aspirated from the joint. Joint aspiration is of prime importance in order to rule out infection.

## Conclusion

This is an uncommon and unusual case of gout in the wrist which occurred in isolation and which may have been induced by a change in anti-hypertensive medication dosage. This case demonstrates several issues that clinicians should keep in mind when assessing patients with a history of gout. Patient education is very important and patients who have had a previous attack of gout should be made aware of common signs and symptoms, treatment protocols during an acute attack, and that gout may occur in any joint of the body, not only in the lower limb. Clinicians should be aware of the various comorbidities associated with gout which include hypertension, cardiovascular disease, and diabetes. Awareness of prescribed medications and any dosage changes is important due to the effects they may have on serum urate levels. Patients should be made aware that dosage changes of certain drugs may precipitate a gouty attack as well as bringing to their attention the effect of aspirin on serum urate levels. Awareness of radiographic changes associated with gout is still of importance although these changes are not seen as frequently as they have been in the past due to better control of the disease.

## Competing interests

The author(s) declare that they have no competing interests.

## Authors' contributions

CLJ and PJS both contributed substantially to the conception, writing and editing of the manuscript. Both authors read and approved the final manuscript.
